# Soil Water Holding Capacity Mitigates Downside Risk and Volatility in US Rainfed Maize: Time to Invest in Soil Organic Matter?

**DOI:** 10.1371/journal.pone.0160974

**Published:** 2016-08-25

**Authors:** Alwyn Williams, Mitchell C. Hunter, Melanie Kammerer, Daniel A. Kane, Nicholas R. Jordan, David A. Mortensen, Richard G. Smith, Sieglinde Snapp, Adam S. Davis

**Affiliations:** 1 Department of Agronomy and Plant Genetics, University of Minnesota, St. Paul, Minnesota, United States of America; 2 Department of Plant Science, Pennsylvania State University, University Park, Pennsylvania, United States of America; 3 Department of Plant, Soil and Microbial Sciences, Michigan State University, East Lansing, Michigan, United States of America; 4 Department of Natural Resources and the Environment, University of New Hampshire, Durham, New Hampshire, United States of America; 5 United States Department of Agriculture-Agricultural Research Service, Global Change and Photosynthesis Research Unit, Urbana, Illinois, United States of America; Instituto Agricultura Sostenible, SPAIN

## Abstract

Yield stability is fundamental to global food security in the face of climate change, and better strategies are needed for buffering crop yields against increased weather variability. Regional- scale analyses of yield stability can support robust inferences about buffering strategies for widely-grown staple crops, but have not been accomplished. We present a novel analytical approach, synthesizing 2000–2014 data on weather and soil factors to quantify their impact on county-level maize yield stability in four US states that vary widely in these factors (Illinois, Michigan, Minnesota and Pennsylvania). Yield stability is quantified as both ‘downside risk’ (minimum yield potential, MYP) and ‘volatility’ (temporal yield variability). We show that excessive heat and drought decreased mean yields and yield stability, while higher precipitation increased stability. Soil water holding capacity strongly affected yield volatility in all four states, either directly (Minnesota and Pennsylvania) or indirectly, via its effects on MYP (Illinois and Michigan). We infer that factors contributing to soil water holding capacity can help buffer maize yields against variable weather. Given that soil water holding capacity responds (within limits) to agronomic management, our analysis highlights broadly relevant management strategies for buffering crop yields against climate variability, and informs region-specific strategies.

## Introduction

Climate change models predict increases in extreme weather over the coming decades (e.g., rising incidence of excessive heat, more intense droughts, and greater frequency of severe rainfall events), with negative consequences for agricultural productivity and yield stability [1–8]. Expected changes in annual rainfall patterns and evapotranspirative demand are forecast to increase drought frequency in many regions, further exacerbating heat stress from warming. This highlights the importance of soil water supply to successful crop production [9, 10], reflected in calls for agronomic management to improve soil function (e.g., by increasing organic matter to increase soil water holding capacity, WHC). It is widely assumed that improvements in these attributes will help buffer crop yields against climate variability [3, 4]. However, few empirical studies have tested this assumption [11]. While improvements to soil attributes have been associated with beneficial effects on crop yields and yield stability in regions where drought and soil degradation are prevalent [12, 13], underlying mechanisms remain unclear, and the scope of inference remains highly limited. Evidence that permits broad inferences is urgently needed to understand the role of soils and their management in agricultural climate adaptation.

Here, we provide such evidence, analyzing joint effects of climate and edaphic factors on yield stability at spatial and temporal scales that subsume finer-grained variation in these factors [14]. Borrowing concepts from finance, namely ‘downside risk’ and ‘volatility’, we quantified yield stability along these two dimensions. Downside risk provides an estimate of the potential for maize yields to decline with adverse environmental conditions, e.g., drought. Volatility provides a measure of maize yield variability over a period of time. Our results highlight how soil properties contribute to regional-scale variation in agricultural climate adaptation. We compiled publicly available data covering the period 2000–2014 to assess the relative contributions of climate and edaphic factors to county-level maize (*Zea mays* L.) yields in four US states with contrasting climates and soil types: Illinois, Michigan, Minnesota and Pennsylvania. Long-term and spatially distributed data on climate, soil and crop yield enable analysis of the interactions among these factors across a range of conditions [15]. Data sources and aggregation procedures are described in Methods. Annual growing season weather data (March through October) included county-level means of daily minimum, maximum and average air temperatures, and cumulative precipitation.

## Results and Discussion

**Fig 1:**
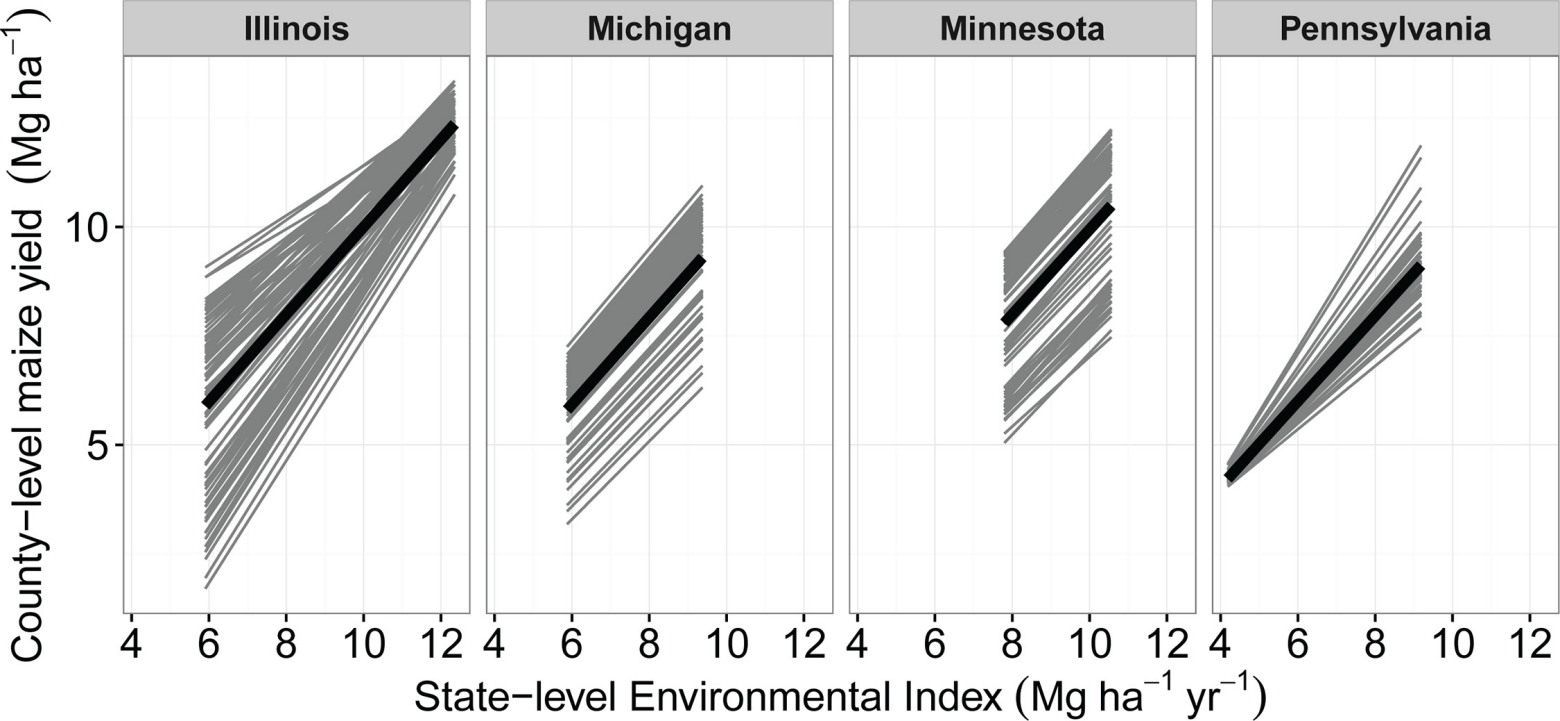
Linear mixed effects models of county-level maize yields (2000–2014) against statewide annual mean of maize grain yield (environmental index). Thick black lines show state mean fixed effect. Gray lines show individual county relationships (best linear unbiased predictors, BLUPs), as estimated from the random effects structure. Number of observations, by state: N_IL_ = 1215, N_MI_ = 705, N_MN_ = 780, N_PA_ = 840.

To quantify downside risk, we used adaptability analysis, in which statewide annual yield averages are used to create an environmental index (EI) that can rank counties and years from ‘poor’ to ‘good’ [16]. County-level maize yield responses to variation in EI were constructed for each state using linear mixed effects models (LMEs) (Fig 1). Variability in EI was driven by fluctuations in maximum temperature and geographic extent of drought (S1 and S2 Figs). In Pennsylvania, the lowest yielding, high elevation counties were also the least drought prone (S1 Table), thus constraining yield variation in drought years. Conversely, low yielding counties in southern Illinois were also the most drought-prone, increasing yield variation in drought years. County-level yields thus converged at maximum EI in Illinois and at minimum EI in Pennsylvania (Fig 1).

LME model predictions at minimum EI values were used to generate minimum yield potential (MYP) values for all counties in each state, which provide a measure of downside risk. A high MYP implies that a county maintained high maize yields under the poorest state-wide environmental conditions (small downside risk); a low MYP indicates that yields were poor during unfavorable environmental conditions (large downside risk). Variation in MYP differed among the four states (S3 Fig). Illinois had a wide range of MYPs, indicating downside risk was large in some counties and small in others. In contrast, counties in Pennsylvania, which generally had the lowest yields under even the most favorable environmental conditions (Fig 1), had a much narrower range of MYPs, i.e. all counties had a similar level of downside risk.

**Fig 2:**
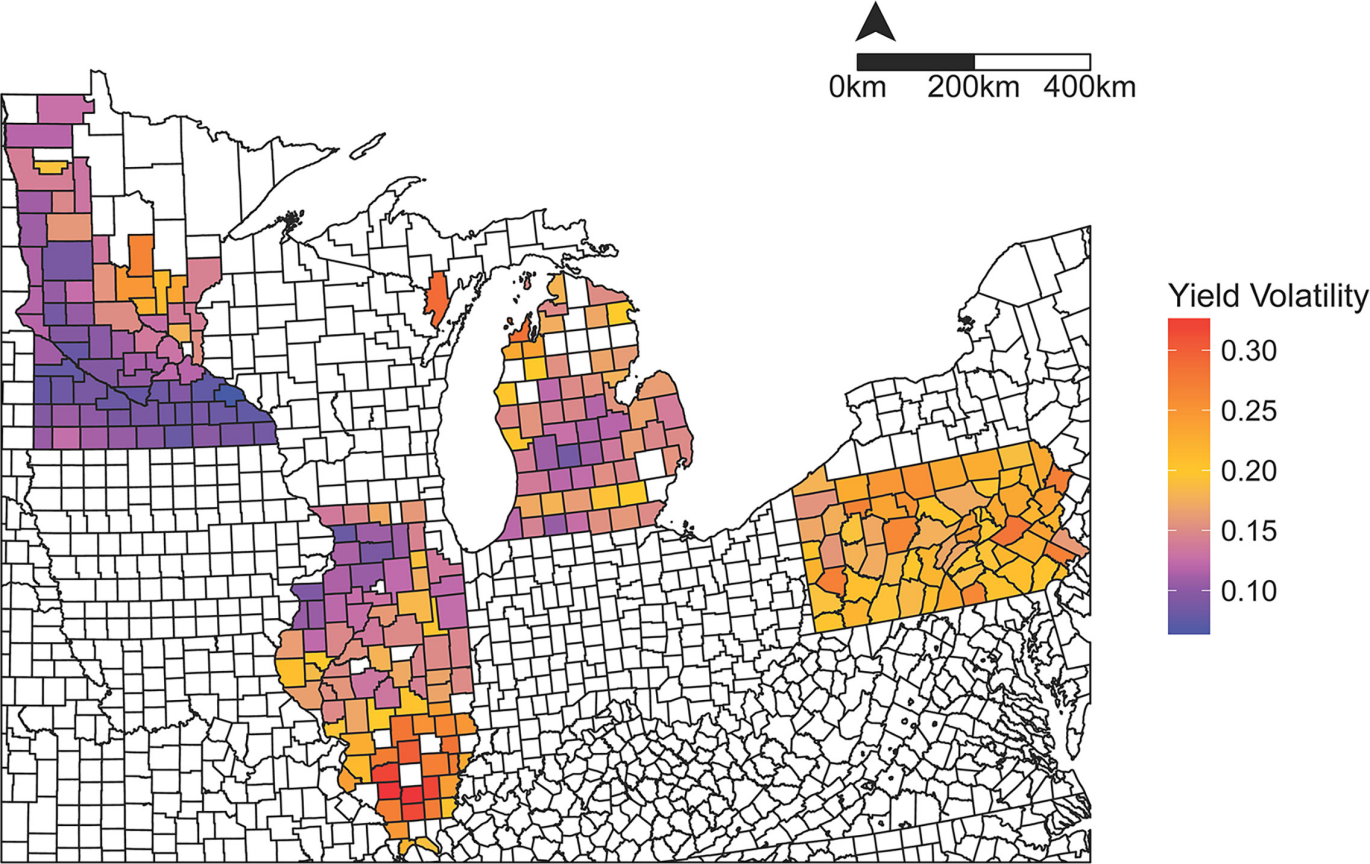
County-level maize yield volatility (coefficient of variation) in each state over 2000–2014. Minnesota (top left state) had the least yield volatility and Pennsylvania (far right) the most. Illinois (second from left) had the greatest variation in yield volatility.

To quantify volatility, we assessed three different measures of county-level temporal yield variability: 1) LME regression slopes [17]; 2) coefficient of variation (CV) [18]; and 3) Power Law Residuals (POLAR) [19]. POLAR are the residuals of the log-log relationship of county-level yield variance against EI, and have been demonstrated to be a useful metric of crop yield stability in some cases [19]. LME regression slopes and POLAR both proved unreliable estimators of volatility, showing inconsistent relationships with MYP, climate, or edaphic variables. CV proved most reliable, and correlated positively with POLAR (S4 Fig). County-level volatility differed greatly among and within states (Fig 2). Overall, maize yields in Minnesota were least volatile, especially in southern counties, while those in Pennsylvania were the most volatile. Within Illinois, there was a strong north-south gradient of increasing yield volatility.

**Fig 3:**
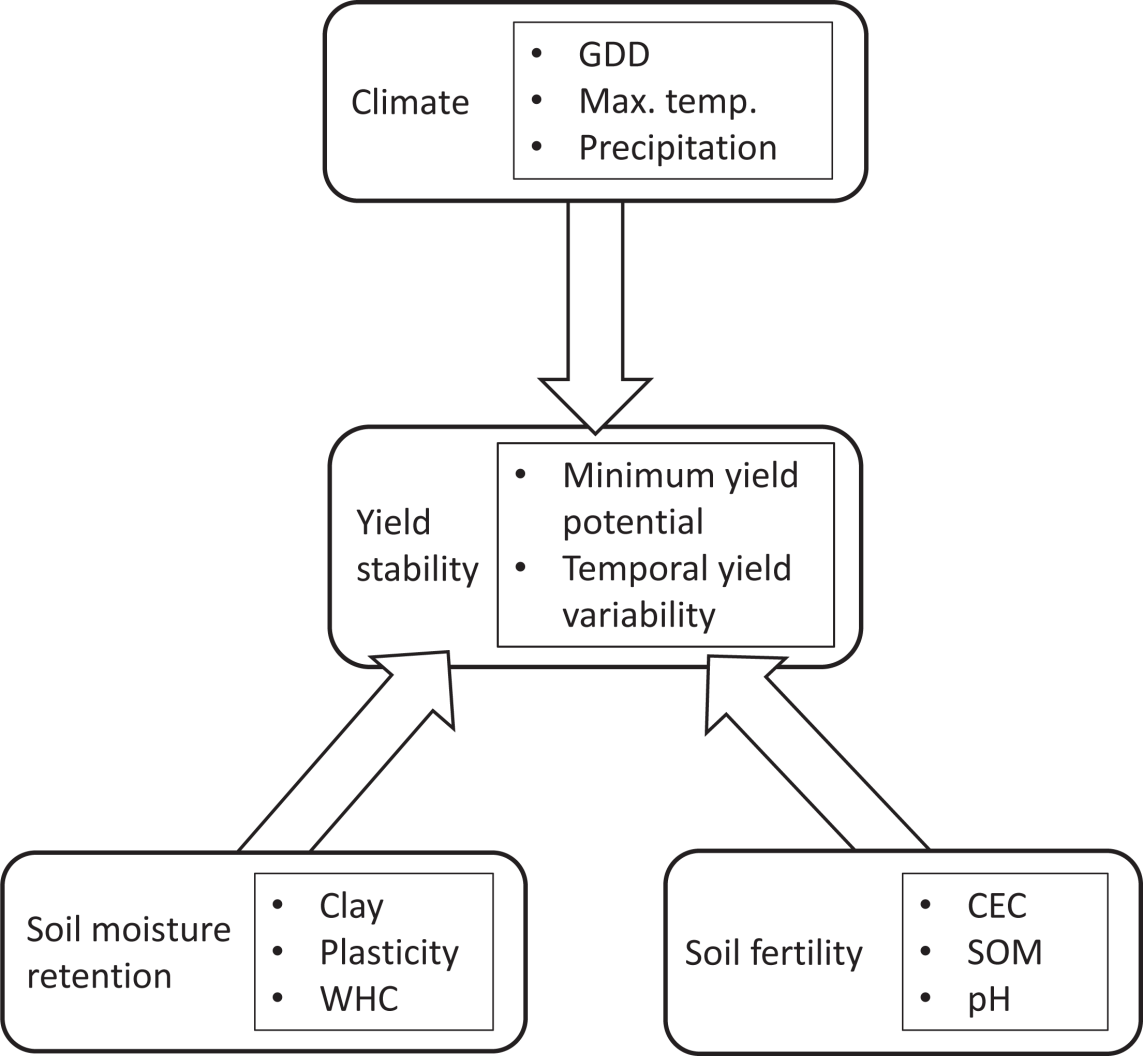
Conceptual framework underpinning structural equation modeling approach to investigate effects of climate and edaphic factors on maize yield stability. Climate data was restricted to growing season periods. Edaphic variables were separated into two components representing complementary soil properties: soil fertility and soil moisture retention.

**Fig 4:**
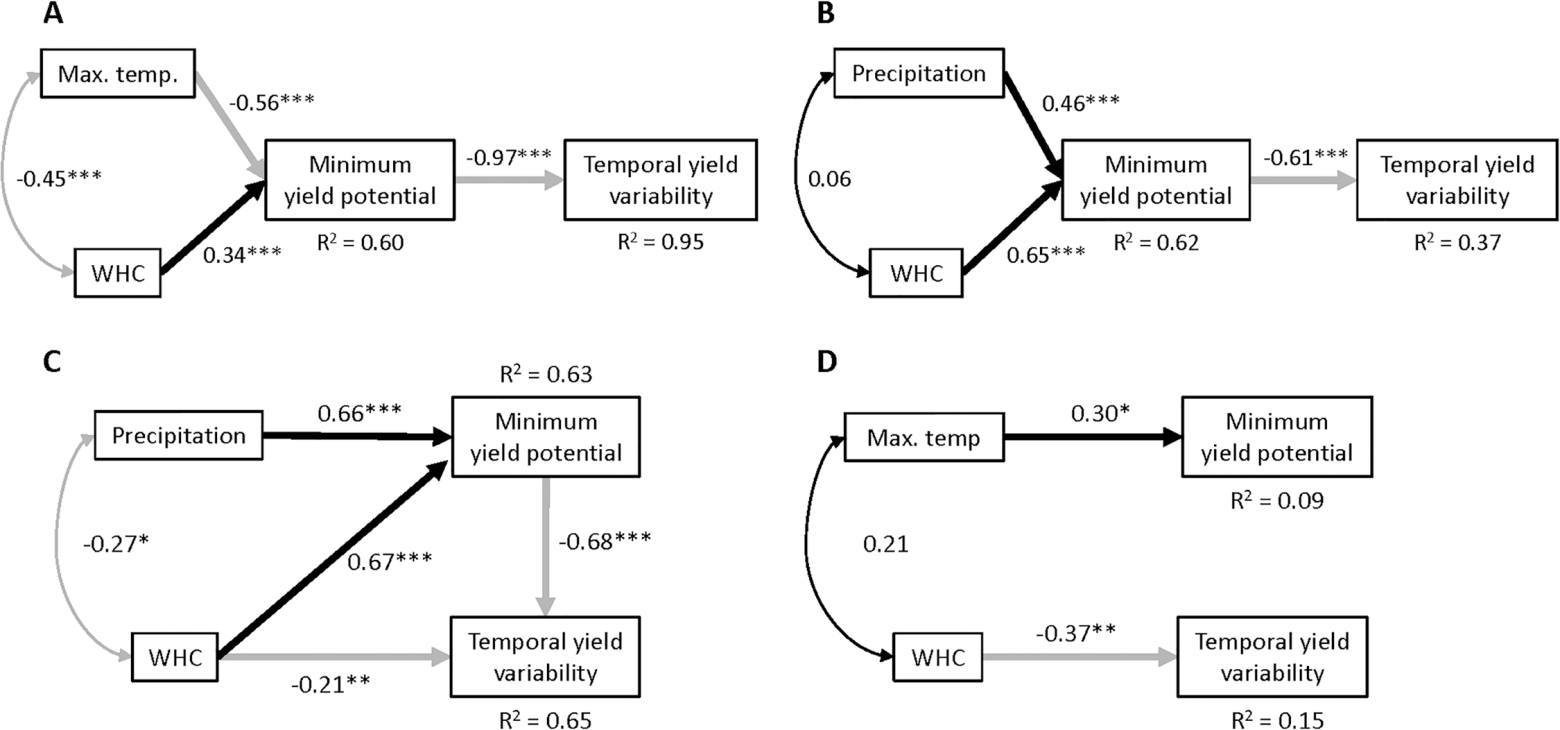
Best supported structural equation models showing effects of climate and edaphic factors on county-level maize minimum yield potential (downside risk) and temporal variability (volatility) for each state. (A) Illinois (χ^2^ = 5.2, df = 2, *P* = 0.07, N = 81), (B) Michigan (χ^2^ = 2.8, df = 2, *P* = 0.24, N = 47), (C) Minnesota (χ^2^ = 0.68, df = 1, *P* = 0.41, N = 52), (D) Pennsylvania (χ^2^ = 0.75, df = 2, *P* = 0.69, N = 56). Model fit values indicate good support for each model. Double-headed arrows denote covariances among variables, whereas single-headed arrows denote standardized regression coefficients. Black arrows show positive relationships; grey arrows show negative relationships. *P* < 0.05 (*), *P* < 0.01 (**), *P* < 0.001 (***).

County-level MYPs and CVs were then incorporated into state-wide structural equation models (SEM, Methods) to assess the relative contributions of climate and edaphic factors to maize production downside risk and yield volatility. The same conceptual framework was applied to each state (Fig 3), and models were simplified using maximum likelihood to find the most parsimonious state-specific model (Fig 4). This conceptual framework hypothesizes that relatively stable soil properties related to moisture retention (percent clay, plasticity index, and water holding capacity) and soil fertility (cation exchange capacity, pH and soil organic matter) interact with weather conditions to affect yield stability.

For Illinois, MYP was positively associated with soil WHC, and negatively associated with average daily maximum growing-season temperatures. In contrast, MYP in Pennsylvania was positively associated with average daily maximum growing-season temperatures. Semi-partial correlations among MYP, maximum temperature and elevation indicated that this association was a signature of better yields in low-lying compared to higher elevation counties in Pennsylvania (r_MYP,maxT| elevation_ = 0.02, P = 0.86; r_MYP, elevation| maxT_ = -0.28, P = 0.03). In both Michigan and Minnesota, increasing levels of precipitation and soil WHC were positively associated with MYP. Higher MYP was associated with reduced yield volatility (temporal yield variability) in Illinois, Michigan and Minnesota, constraining the range within which yield variation could occur. In Minnesota and Pennsylvania, yield volatility also declined directly with increasing WHC. No relationship was observed between MYP and yield volatility in Pennsylvania, likely due to low variation in MYP (Fig 1 and S3 Fig). Although SOM, CEC, percent clay and pH were not retained in the best supported SEM models, they showed positive associations with maize yield stability in IL, MI and MN and negative associations with maximum drought extent in all four states (S1 Table).

The results show strong regional influences of climate and edaphic factors on maize yield stability across 2000–2014. Our findings extend insights from previous studies that showed negative impacts of extreme temperatures and drought on maize yields [14, 15, 20]. Our findings also reveal substantial regional variation in climate and edaphic factors on maize yield stability. Recent studies indicate that the negative effect of high summer temperatures is due less to effects on reproductive growth (e.g., heat damage between anthesis and silking reducing pollen and grain set) and more to increased moisture stress driven by vapor pressure deficit (VPD) [15, 21]. Rising VPD increases evapotranspiration, which has a two-fold impact on crop moisture stress: 1) photosynthesis declines as crops that are unable to meet transpirative demand reduce their stomatal conductance and 2) soil water supply to the crop declines due to increased evaporation from the soil surface [20].

Soil WHC had a positive effect on yield stability in all states (Fig 4), either through direct reductions in yield volatility (Minnesota, Pennsylvania) or by limiting downside risk (Illinois, Michigan, Minnesota). In addition, precipitation, which a previous study found to have a weak effect on crop yields in Iowa [21], correlated positively with MYP in both Michigan and Minnesota (Fig 4). These results, in conjunction with maximum summer temperature data, all point to strong maize yield sensitivity to increases in VPD [15], and demonstrate the importance of maintaining adequate soil moisture to meet crop water demand. WHC in Illinois increased with latitude (r = 0.43, P< 0.0001), underlying the negative association between maximum temperature and WHC. Across states, semi-partial correlations among soil WHC, clay content and SOM (r _WHC,clay|SOM_ = 0.92, P< 0.0001; r _WHC,SOM|clay_ = 0.58, P< 0.0001) showed that although soil texture was strongly associated with variation in WHC, SOM made substantial contributions to WHC variation that were distinct from those of soil clay content.

In totality, these results suggest a novel hypothesis that requires testing at landscape to regional scales: US maize producers should manage soils to increase SOM and thereby improve yield stability. Within pedogenic constraints on SOM accrual and aggregate formation, agricultural management that increases SOM may increase WHC and thereby the soil’s capacity to meet crop water demand. Indeed, previous work in China has shown that cereal productivity and yield stability are strongly correlated with SOM [11]. SOM holds water directly and also helps bind soil aggregates, which improves soil structure and WHC while also enhancing rainfall infiltration [22, 23]. Thus, changes to agronomic management that encourage SOM accumulation may increase soil WHC to counter rising VPD, and thereby increase yield stability. Such management changes include reducing tillage, managing residues, and increasing organic matter inputs through use of compost, manure or cover crops [24, 25, 26].

Our analysis quantifies the interacting effects of soil properties and weather variability on rainfed maize yield stability in the US over 2000–2014. The results portend substantial yield losses and an increase in yield instability as summer temperatures and precipitation shortfalls increase in frequency due to climate change [1, 2]. The consistent stabilizing impact of soil WHC found across all four States indicates that soil attributes responsive to agronomic management may strongly mediate the effects of climate change on maize yield stability, due to the importance of meeting crop water demand [15, 21]. This suggests that relatively simple changes to agricultural soil management aimed at enhancing SOM can contribute meaningfully to climate adaptation by increasing WHC [3, 25]. However, the scope for improving SOM and WHC is constrained by soil type [27, 28], so some regions may have limited opportunities to mitigate climate change impacts through improved soil management. Other strategies will be required to complement WHC increases, such as crop genetic improvement, cropping system design, and irrigation technologies, among others [29]. The analytical approach developed here can be used to better inform agricultural climate adaptation strategies, and help guide the selective deployment of a broad suite of regionally-specific climate adaptation strategies.

## Methods

### Database aggregation

County-level data for US maize yields, soil chemical and physical properties, drought status and weather were downloaded from public databases for the 2000 through 2014 period. Maize annual yield data were from the US Department of Agriculture (USDA) National Agricultural Statistics Service [30]; soils data from the USDA Web Soil Survey [31]; drought status from the USDA National Drought Mitigation Center [32] and weather data from the National Oceanic and Atmospheric Administration (NOAA) National Centers for Environmental Information [33]. Counties with fewer than 10-years of complete data over 2000–2014 were discarded.

Soil chemical properties from the Web Soil Survey included cation exchange capacity (CEC; meq 100 g soil^-1^ at pH 7), pH (1:1 water:soil method), soil organic matter (SOM; %), and soil physical properties included clay content (%), plasticity index (%; the range of soil moisture contents in which a soil exhibits the properties of a plastic solid) and water holding capacity at 15 bar (WHC; % v/v). Only soils being used for agricultural purposes (‘cultivated’ land as defined by the USDA National Agricultural Statistics Service 2014 Cultivated Layer [34]) were included in the dataset. Soil properties were calculated as an area-weighted average across soil types, over 0 to 30 cm depth (the ‘plow layer’) [31].

County-level drought data were obtained as within-year season-long (April through September) averages of the weekly percentage of crop land within a county classified as experiencing moderate (D1) to exceptional (D4) drought intensity [32]. To examine the relationship between county-level soil and site variables and drought intensity within each state, we compiled a variable ‘D1.D4.worst’, which represented the percentage of area in each county experiencing drought intensity of classes D1 (moderate) to D4 (exceptional) in the worst drought year for each state (highest overall percentage of state in classes D1 to D4) during the study period. During 2000–2014, Illinois experienced its most severe level of drought in 2012, Michigan and Minnesota in 2007, and Pennsylvania in 2002.

County-level annual weather data were obtained as a within-year average of data from all weather stations present in the NOAA data set for a given county. Weather data included daily minimum and maximum air temperatures and precipitation for the period from March through October. From these data, we calculated season-long county averages for maximum, minimum and mean temperatures as well as cumulative growing degree days, which were calculated as follows:

$${GDD}_{i}=\sum_{j=1}^{n}\frac{T_{max}+T_{min}}{2}-T_{b}$$ [1]

where *T*_*max*_ and *T*_*min*_ are maximum and minimum air temperatures (°C), respectively, and *T*_*b*_ is the base temperature for calculating thermal time for maize (10°C), with the summation performed for county *i* for the *j*^*th*^ day from March through October, summing only those daily GDD values > 0.

### Data analysis

Traditional adaptability analysis, or Finlay-Wilkinson regression, quantifies treatment-specific variation in yield response to variation in environmental suitability, represented as the mean yield across treatments across a range of environments (EI, the Environmental Index) [16, 17]. In our analysis, we utilized the adaptability analysis approach to analyze annual county-level maize yields for each of our four states over 2000–2014 in relation to variation in each state’s annual mean yield over 2000–2014; for each state, annual mean state-wide yields were used as our EI. We implemented adaptability analysis by fitting linear mixed effects models (LMEs) for each state, using the *nlme* package of R 3.2.2 [35]. Each model contained a county-level random intercept and slope structure, and EI was fitted as the fixed effect. Within R, this gave the following model structure: county-level maize yield ~ EI, random = ~ EI | county. County-level minimum yield potential (MYP; ‘downside risk’) values were calculated from LME predictions of maize yield at the minimum EI for each state. Coefficient of variation (CV; ‘yield volatility’) was calculated for each county within a state as the quotient of maize yield standard deviation over mean maize yield.

Global state-wide structural equation models were organized according to Fig 3, with individual climate, soil fertility and soil moisture retention variables grouped into corresponding latent variables [36]. SEM models allowed us to quantify the contributions of these variables to maize production downside risk and yield volatility, while accounting for spatiotemporal covariances among them. A candidate pool of SEM models containing the global model and more parsimonious subsets of this model, with soil properties and climate represented by either latent variables, manifest variables, or both was fit using the *lavaan* package of R 3.2.2 [35]. These candidate models were discarded or retained using maximum likelihood estimations and Akaike (AIC) weights to find the most parsimonious models. In addition, models were assessed for ‘goodness of fit’ using χ^2^-values, comparative fit index (CFI), and root mean square error of approximation (RMSEA) [36].

## Supporting Information

S1 Fig BLUPs (best linear unbiased predictors) from linear mixed effects model quantifying state-level associations between Environmental Index (annual mean of maize yield in Mg ha^-1^) and statewide mean of maximum daily temperature (°C). Fixed effects: intercept = 19.56 (t_1, 55_ = 4.2, P < 0.001), maximum temperature = -0.44 (t_1, 55_ = -2.49, P = 0.016). Random effects: standard deviation (maximum temperature) = 0.19, standard deviation (residual) = 1.04. N = 15 for all states.
(EPS)

S2 Fig BLUPs (best linear unbiased predictors) from linear mixed effects model quantifying state-level associations between Environmental Index (annual mean of maize yield in Mg ha^-1^) and statewide percent area in drought classes D1 to D4 (%)^31^. Fixed effects: intercept = 9.6 (t_1, 55_ = 19.6, P < 0.0001), % area in drought class = -0.06 (t_1, 232_ = -2.1, P = 0.04). Random effects: standard deviation (% area in drought class) = 0.05, standard deviation (residual) = 0.88. N = 15 for all states.
(EPS)

S3 Fig Box-plots showing distributions of MYP (minimum yield potential), represented by county-level predictions at the lowest value Environmental Index (EI: statewide annual mean of maize grain yield) from linear mixed effects models of county-level maize yield response to variation in EI. Number of observations, by state: N_IL_ = 84, N_MI_ = 47, N_MN_ = 52, N_PA_ = 56.
(EPS)

S4 Fig BLUPs (best linear unbiased predictors) from linear mixed effects model quantifying state-level associations between two measures of maize yield stability: CV (coefficient of variation) and POLAR (Power Law Residuals). Fixed effects: intercept = 0.17 (t_1, 232_ = 10.3, P < 0.0001), POLAR = 0.18 (t_1, 232_ = 8.2, P < 0.0001). Random effects: standard deviation (POLAR) = 0.023, standard deviation (residual) = 0.04. Number of observations, by state: N_IL_ = 84, N_MI_ = 47, N_MN_ = 52, N_PA_ = 56.
(EPS)

S1 Table Pearson correlations between county-level indices of maize yield stability and drought and selected soil and site properties in four maize-producing US states. (PDF)
