# Supplementary material for: Soil Water Holding Capacity Mitigates Downside Risk and Volatility in US Rainfed Maize: Time to Invest in Soil Organic Matter?
Source: PLoS One. 2016 Aug 25;11(8):e0160974. doi: 10.1371/journal.pone.0160974 (PMC4999238; doi:10.1371/journal.pone.0160974)
Supplement: S1 Table — (PDF) [file pone.0160974.s005.pdf]

S1 Table. Pearson correlations between county-level indices of maize yield stability and drought and selected soil and site properties in four maize-producing US states

State	County indices[a]	Soil properties[b]					Site properties	
		WHC	SOM	CEC	pH	clay	elevation	latitude
						Pearson correlations (r)		
Illinois	CV	-0.56 ***	-0.69 ***	-0.70 ***	-0.62 ***	-0.42 **	-0.74 ***	-0.80 ***
	MYP	0.59 ***	0.70 ***	0.72 ***	0.55 ***	0.45 **	0.75 ***	0.78 ***
	D1.D4	-0.42 ***	-0.52 ***	-0.45 ***	-0.53 ***	-0.21	-0.51 ***	-0.57 ***
Michigan	CV	-0.33 *	-0.17	-0.27	-0.09	-0.32 *	-0.01	0.46 ***
	MYP	0.66 ***	0.35 *	0.41 **	0.44 **	0.61 ***	-0.15	-0.55 ***
	D1.D4	-0.54 ***	-0.36 *	-0.25	-0.31 *	-0.49 ***	0.09	0.31 *
Minnesota	CV	-0.54 ***	-0.08	-0.45 **	-0.48 ***	-0.60 ***	0.09	0.47 ***
	MYP	0.48 ***	-0.01	0.35 *	0.19	0.56 ***	-0.20	-0.85 ***
	D1.D4	-0.47 ***	0.11	-0.43 **	-0.51 ***	-0.53 ***	-0.01	-0.04
Pennsylvania	CV	-0.37**	0.09	-0.15	-0.16	-0.19	0.02	0.05
	MYP	0.15	-0.18	0.08	0.15	0.22	-0.40 **	-0.23
	D1.D4	0.05	-0.26 *	-0.32 *	-0.39 *	0.19	-0.76 ***	-0.60 ***

[a] Explanation of county-level indices of maize yield stability and drought susceptibility: CV = coefficient of variation in county-level maize yield over the study period (volatility); MYP = minimum county-level yield potential (downside risk); D1.D4 = percent of county in USDA drought classes D1 to D4 in 2012, the most severe drought during the 2000 to 2014 period.

[b] Soil property abbreviations: WHC = water holding capacity at 15 bar (% volumetric soil moisture); SOM = soil organic matter (%); CEC = cation exchange capacity (meq 100 g $soil^{-1}$); clay = % clay content

[c] The symbols *, ** and *** denote significant correlations between yield stability and a given soil property at $P < 0.05$, $P < 0.01$ and $P < 0.001$, respectively.
